# Septicemic Melioidosis Detection Using Support Vector Machine with Five Immune Cell Types

**DOI:** 10.1155/2021/8668978

**Published:** 2021-12-06

**Authors:** Ke Xu, Fang Lian, Yunfan Quan, Jun Liu, Li Yin, Xuexia Li, Shen Tian, Hua Pei, Qianfeng Xia

**Affiliations:** ^1^Key Laboratory of Tropical Translational Medicine of Ministry of Education and School of Tropical Medicine and Laboratory Medicine, Hainan Medical University, Haikou, Hainan, China; ^2^Department of Clinical Laboratory, The Second Affiliated Hospital, Hainan Medical University, Haikou, China; ^3^School of Basic Medicine and Life Sciences, Hainan Medical University, Haikou, Hainan, China

## Abstract

Melioidosis, caused by *Burkholderia pseudomallei* (*B. pseudomallei*), predominantly occurs in the tropical regions. Of various types of melioidosis, septicemic melioidosis is the most lethal one with a mortality rate of 40%. Early detection of the disease is paramount for the better chances of cure. In this study, we developed a novel approach for septicemic melioidosis detection, using a machine learning technique—support vector machine (SVM). Several SVM models were built, and 19 features characterized by the corresponding immune cell types were generated by Cell type Identification Estimating Relative Subsets Of RNA Transcripts (CIBERSORT). Using these features, we trained a binomial SVM model on the training set and evaluated it on the independent testing set. Our findings indicated that this model performed well with means of sensitivity and specificity up to 0.962 and 0.979, respectively. Meanwhile, the receiver operating characteristic (ROC) curve analysis gave area under curves (AUCs) ranging from 0.952 to 1.000. Furthermore, we found that a concise SVM model, built upon a combination of CD8+ T cells, resting CD4+ memory T cells, monocytes, M2 macrophages, and activated mast cells, worked perfectly on the detection of septicemic melioidosis. Our data showed that its mean of sensitivity was up to 0.976 while that of specificity up to 0.993. In addition, the ROC curve analysis gave AUC close to 1.000. Taken together, this SVM model is a robust classification tool and may serve as a complementary diagnostic technique to septicemic melioidosis.

## 1. Introduction

Melioidosis is a serious tropical infectious disease that frequently outbreaks in Southeast Asia and northern Australia. The disease is caused by the gram-negative bacillus *Burkholderia pseudomallei* (*B. pseudomallei*), which inhabits in soil and surface water [1]. In endemic regions, melioidosis is a main cause of lethal community-acquired septicemia and pneumonia in adults [2, 3], and mortality rates range from10 to 50% [4]. Biologically, *B. pseudomallei* first invades epithelial cells of the mucosal surface or broken skin, is expanded intracellularly, and then disseminates to a variety of cell types, resulting in bacteremia and sapremia [5]. Basically, the *B. pseudomallei* infection leads to two types of melioidosis, acute melioidosis and chronic melioidosis. Of note, nearly 85% of melioidosis cases are of acute type, and patients with acute melioidosis develop sepsis rapidly, namely, septicemic melioidosis. This condition is a life-threatening with a mortality rate of 40% [6].

High mortality rate of patients with acute melioidosis is attributed to limitations on early clinical diagnosis. A case in point is that the bacterial blood culture remains the mainstay of melioidosis diagnosis. In fact, *B. pseudomallei* can grow on the culture media, but its growth is extremely slower than other microbes. In some cases, it takes up to more than four days for the establishment of bacterial colonies. That may miss out the best time for melioidosis treatment, as Tiangpitayakorn et al. reported that 35% of melioidosis patients died within 2 days of admission [7]. The mortality rate of the patients treated with melioidosis-appropriate drug regimens within two days of admission was decreased by 30% [8]. These findings suggest that early detection for melioidosis is fundamental for the better chances of cure and has been recognized by many researchers as a focus of research in melioidosis. As a result, a variety of detection approaches have been developed based upon different theories. Nevertheless, these approaches, which are either performed poorly or costly, are not able to be implemented in the clinical settings [9–11]. This prompts us to search for a new detection approach with fast, accurate, yet inexpensive advantages.

Many patients with melioidosis have abnormal immune conditions, suggesting that the onset and development of the disease are mainly determined by the host's immune status [12–14]. This has been exemplified by twofold. On the one hand, most of immunocompetent individuals can clear the *B. pseudomallei* infection, leaving no clinical symptom [15]. On the other hand, individuals with long-term use of steroid, which leads to the induction and maintenance of immunosuppression, are susceptible to *B. pseudomallei* infection [15]. In addition, upon the bacterial invasion, neutrophils and macrophages were recruited at the site of infection, and neutrophils directly led to the bacterial containment [16]. Also, the T cell-mediated immune response is essential for the prevention of the active infection [17]. Jenjaroen et al. showed that the patients with melioidosis displayed the increased levels of CD4+ and CD8+ T cells, and the levels of these cells were decreased with increased mortality [18]. These abovementioned evidence supports the roles played by neutrophil macrophages and lymphocytes in the onset and development of melioidosis. The changes in the levels of these immune cells reflect the disease status. Thus, a key question is whether the immune cells serving as a unique pattern can be used for the detection of melioidosis.

Peripheral blood is an accessible source of immune cells and reflects the real immune status of individuals. Previous studies have provided useful insight based upon peripheral blood samples subjected to microarray-based profiling and analysis, suggesting that it is an instrumental approach to investigate melioidosis [19].

Computer-aided diagnosis, which are designed to help improve the accuracy of disease diagnosis [20], has been recognized by the World Health Organization (WHO) [21]. Recently, a number of computer-aided diagnoses were performed with the supervised machine learning (SML). Briefly, SML learns known data to build a mathematical model and predicts unknown data [22]. The key process incorporates multiple different statistical, probabilistic, and optimization techniques. Among a variety of learning models, the support vector machine (SVM) is a very powerful and versatile machine learning model, capable of performing linear or nonlinear classification. More importantly, SVMs are particularly well suited for classification of complex but small- or medium-sized datasets [23, 24]. Since the high-throughput microarray gene expression data was available in the early 2000s, researchers have implemented SVM on disease classification. First trial was conducted with an SVM to classify two different types of leukemia using gene expression microarray dataset in 1999 [25]. Since then, a series of breakthroughs have been achieved in clinical diagnoses [26, 27]. In this study, we employed the algorithm “Cell type Identification By Estimating Relative Subsets Of RNA Transcripts (CIBERSORT),” which has been deemed to be the most accurate method available [28]. This algorithm allows us to generate a dataset of 22 immune cell types. This dataset was then input into the training models, and a series of good classifications were made. With a series of feature selections, we found that a novel SVM model, trained by a combination of CD8+ T cells, resting CD4+ memory T cells, monocytes, M2 macrophages, and activated mast cells, is able to achieve accurate detection of septicemic melioidosis.

## 2. Material and Methods

### 2.1. Dataset Collection

The human peripheral blood microarray data were obtained from NCBI GEO datasets, the accession numbers were GSE13015 and GSE69528. In this study, 36 healthy donors (health), 39 patients with type 2 diabetes (type 2 diabetes), and 9 patients who had recovered from melioidosis (recovery), 69 patients with septicemic melioidosis (B.pseudomallei) were grouped. In addition, 91 patients with sepsis due to other organisms (other infections) were grouped, respectively, including 3 Aeromonas spp., 2 Acinetobacter lwoffii, 3 Acinetobacter baumannii, 1 Bacillus spp., Micrococcus spp., 1 Citrobacter freundii, 7 Corynebacterium spp., 1 Cryptococcus neoformans, 4 Enterococcus spp., 16 E. coli, 1 Escherichia coli, Viridans streptococcus, 1 Klebsiella pneumoniae, Viridans streptococci, 1 Klebsiella spp., Pseudomonas aeruginosa, 6 Klebsiella pneumonia, 4 Micrococcus spp., 4 Salmonella, 1 Salmonella gr. C, 1 Sphingobacterium spp., 1 Sphingomonas spp., 9 Staphylococcus aureus, 1 Streptococcus suis, 3 Streptococcus pyogenes, 4 Viridans streptococci, 1 Acinetobacter baumannii, 4 C. albicans, 1 Enterococcus faecium, 1 S. pneumoniae, 1 Salmonella serotype B, 6 Staphylococcus coagulase negative, and 2 Streptococcus non A, B.

### 2.2. CIBERSORT Analysis

CIBERSORT, as an instrumental tool, can precisely measure the relative levels of distinct immune cell types. It can characterize each immune cell type with a bulk gene expression signature consisting of 500 genes or so. The original CIBERSORT gene signature file LM22, defining 22 immune cell types, was applied for analyzing the dataset from the septicemic melioidosis. CIBERSORT metrics with pearson correlation coefficient, CIBERSORT *p* value, and root mean squared error (RMSE) were measured for each sample [28].

### 2.3. Training and Testing Set

Features and corresponding labels determining septicemic melioidosis status (positive for septicemic melioidosis and negative for no septicemic melioidosis) across all the samples were integrated into a data frame. The data frame was then randomly split into two data sets, the training and testing ones, according to a ratio of seven to three.

### 2.4. Support Vector Machine

We utilized an SVM framework provided by the python package scikit-learn to build our binomial (septicemic melioidosis and no septicemic melioidosis) classifier. As the support vector machine is sensitive to feature scales, our classifier was actually designed to incorporate a two-step framework: the first step was a standardized scaler provided by StandardScaler class of sklearn for feature normalization, and the second step was a support vector machine classifier. A radial basis function (rbf) kernel of SVM was prioritized, and hyperparameters, gamma, and C were fine-tuned on training dataset using grid search paired with threefold cross validation, which was carried out using GridSearchCV function of sklearn. The hyperparameter set yielding the greatest average score through cross-validation was selected. The SVM framework with optimized hyperparameters was then implemented on the independent testing set for final validation.

### 2.5. Feature Selection

We conducted three feature selection methods in our framework. These feature selection techniques include filter-based method (select K Best (SKB)), wrapped method (recursive feature elimination), and embedded-based method (select from model (SM)). The latter two require estimators that have important features or coefficient properties. Random forest was intentionally employed as estimator, as it is eligible and can be manipulated easily.

## 3. Results

### 3.1. Principal Component Analysis Exhibited the Model Data Distributions

**Figure 1:**
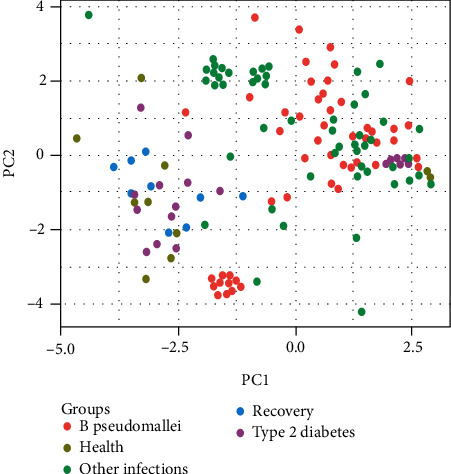
Principal component analysis depicting the model data distributions. B. pseudomallei denotes patients with septicemic melioidosis. Health denotes healthy donors. Type 2 diabetes denotes individuals with type 2 diabetes mellitus but have no *B. pseudomallei* infection. Recover denotes individuals recovering from septicemic melioidosis. Other infections denotes those infected with other bacterial pathogens.

Having conducted CIBERSORT analysis, we obtained a large table with 22 immune cell types. The values of follicular T helper cell, dendritic resting cell, and eosinophil in more than 80% samples are zero, which is empirically useless for analysis. We therefore removed the three cell types. Principal component analysis (PCA) is by far the most popular dimensionality reduction algorithm. It identifies the right hyperplane that lies closest to the data, and the data were then projected onto it. In order to get an intuitive sense of the model data distribution, we conducted the principal component analysis. In Figure 1, the result of the projection of our dataset onto the two dimensional space was displayed. As you viewed that majority of healthy donor (health) instances were aggregated tightly on the middle right area, while the remaining spread far apart over a wide area on the left. These instances were overlapped with those of a small portion of patients with type 2 diabetes (type 2 diabetes) as well as with those of all the patients who had recovered from melioidosis (recovery). We first intended to separate patients with septicemic melioidosis (B. pseudomallei) from health, type 2 diabetes, and recovery. It seems likely that the data is nonlinearly separable since several outliers were located on the opposite side, making it impossible to find a line for separation. To address this challenge, it is preferable to select a more flexible model. Another issue was that parts of instances of B. pseudomallei and patients with sepsis due to other organisms (other infections) were massively overlapped. Tackling this issue might require to add new features (dimensionality increase) using some specialized functions or to remove some outliers.

### 3.2. Performance of Support Vector Machine on the Health, Type 2 Diabetes, and Recovery Dataset

**Figure 2:**
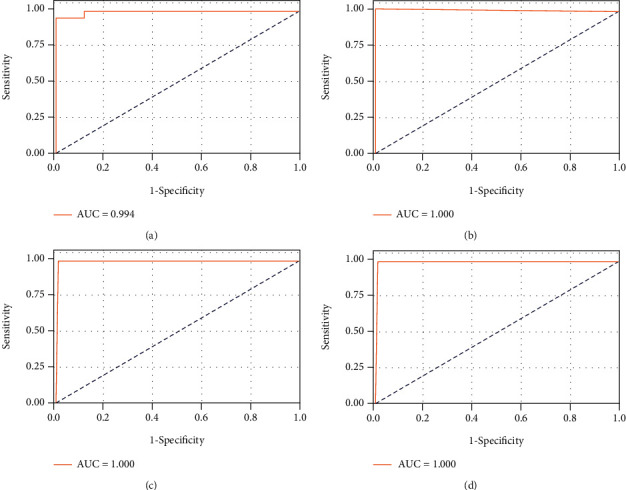
Receiver operating characteristic curves depicting SVM classification between patients with septicemic melioidosis and a mixed group including healthy donors, individuals with type 2 diabetes mellitus, and individuals recovering from septicemic melioidosis on testing dataset. Four subplots represent randomly resampling four times. AUC: area under the curve. Sensitivity and specificity were shown in the following brackets: (a) (0.994, 0.952, 1.000); (b) (1.000, 1.000, 1.000); (c) (1.000, 1.000, 1.000); (d) (1.000, 1.000, 1.000).

153 instances including 36 health, 39 type 2 diabetes, 69 B. pseudomallei, and 9 recovery, with 19 features, were randomly split into two datasets, the training and testing using 7 : 3 ratio. Accordingly, the training set incorporated 107 instances, and the testing set contained 46 instances. SVM classifier with a radical basis function (rbf) kernel in concert with default hyperparameters was implemented on the training set. After that, the trained model was performed on the testing dataset. This process was repeated four times, in order to obtain a reliable evaluation for the model. As predicted, B. pseudomallei and a mixed group including health, type 2 diabetes, and recovery were well separated (Figure 2). The means of sensitivity and specificity reach up to 0.988 and 1.000, respectively.

### 3.3. Performance of Support Vector Machine on the Other Infection Dataset

**Figure 3:**
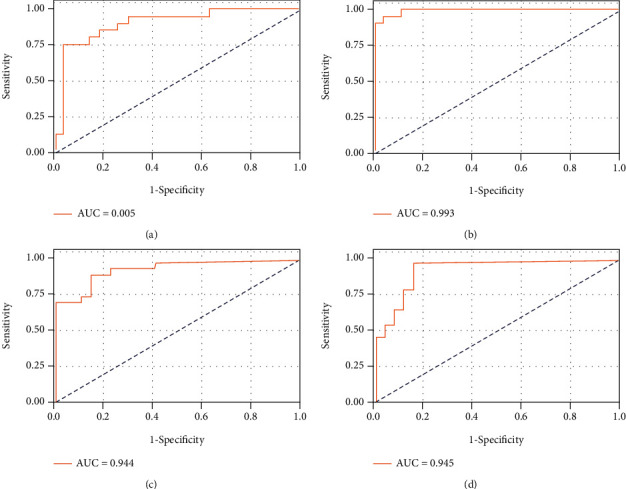
Receiver operating characteristic curves depicting SVM classification between patients with septicemic melioidosis and other infections on testing dataset. Four subplots represent randomly resampling four times. AUC: area under the curve. Sensitivity and specificity were shown in the following brackets: (a) (0.905, 0.857, 0.889); (b) (0.993, 1.000, 1.000); (c) (0.944, 0.905, 1.000); (d) (0.945, 0.905, 1.000).

**Figure 4:**
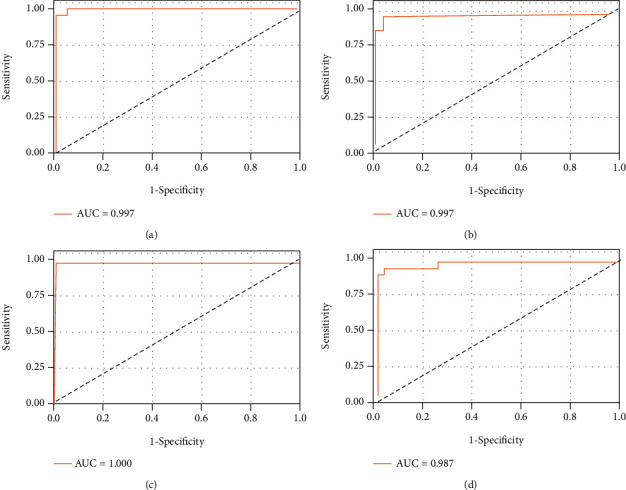
Receiver operating characteristic curves depicting SVM classification between patients with septicemic melioidosis and a mixed group including healthy donors, individuals with type 2 diabetes mellitus, individuals recovering from septicemic melioidosis, and part of other infections on testing dataset. Four subplots represent randomly resampling four times. AUC: area under the curve. Sensitivity and specificity were shown in the following brackets: (a) (0.997, 0.952, 1.000); (b) (0.997, 0.952, 0.972); (c) (1.000, 1.000, 0.972); (d) (0.987, 0.952, 0.972).

160 instances (69 B. pseudomallei and 91 other infections) with 19 features were randomly split into two datasets: training and testing using 7 : 3 ratio. Accordingly, the training set incorporated 112 instances, and the testing set contained 48 instances. An SVM model with an rbf kernel and default hyperparameters were implemented on the training set. After that, the trained model was performed on the testing dataset. This process was repeated four times, in order to obtain a reliable evaluation for the model. As shown in Figure 3, classifying B. pseudomallei from other infections was difficult. The values of sensitivity ranged from 0.857 to 1.000 and those of specificity from 0.889 to 1.000. Since kernel selection is very important for the SVM performance and regularization (namely, tuning of hyperparameters) of the model is likely to further improve its performance, it is possible that a proper manipulation of the model selection and regularization might achieve a better performance. Therefore, we tested whether a particular set of model and hyperparameters could be selected. However, any attempt to improve the SVM performance failed, suggesting that the dataset per se may be inseparable. We reasoned that removal of some outliers from the dataset might be a better way to improve the model performance. Therefore, some combinations of 29 elements from other infections were generated, and removal of Corynebacterium spp., Enterococcus spp., E.coli, Salmonella, Staphylococcus aureus, Acinetobacter baumannii, C. albicans, Staphylococcus coagulase negative, Streptococcus non A,B, Enterococcus faecium, S. pneumoniae, and Salmonella serotype B indeed improved the model performance according to the model performance score (data not shown). The trimmed dataset was integrated into those of B. pseudomallei, health, type 2 diabetes, and recovery. An SVM model with an rbf kernel was applied on this new table. The data of Figure 4 showed that this SVM model worked perfectly. It achieved the mean of sensitivity of 0.962 and that of specificity of 0.979.

### 3.4. Feature Selections

One intention of our work was to provide useful guidance for the clinical diagnosis of melioidosis. Using flow cytometry to examine 19 immune cell types across the samples is laborious, costly, time-consuming, and, more importantly, infeasible. Besides, some features may be redundant or irrelevant, and they harm the performance of the model. Therefore, it is required to conduct feature selection to remove those redundant and irrelevant. Here, three feature selection techniques including filter-based method (select K Best (SKB)), wrapped method (recursive feature elimination), and embedded-based method (select from model (SM)) were employed. As a result, three sets of features were selected ((1) naïve B cells, plasma cells, gamma delta T cells, resting NK cells, activated NK cells, M0 macrophages, activated dendritic cells, activated mast cells, and neutrophils; (2) naïve B. cells, plasma cells, CD8+ T cells, naïve CD4+ T cells, gamma delta T cells, resting NK cells, activated NK cells, monocytes,. M0 macrophages, activated dendritic cells, activated mast cells, and neutrophils; and (3) naïve B cells, plasma cells, naïve CD4+ T cells, resting CD4+ memory T cells, regulatory T cells, activated NK cells, M0 macrophages, M2 macrophages, activated dendritic cells, resting mast cells, and activated mast cells). The three sets of features were pooled, generating a set of 16 features. We next screened all possible combinations of the 16 features. The entire process was evaluated by the model performance score, and our ultimate goal was to search for an excellent combination with fewer elements. Our findings indicated that CD8+ T cells, resting CD4+ memory T cells, monocytes, M2 macrophages, and activated mast cells were relevant features. Only when these five features worked together could we improve the SVM model (rbf kernel) performance, with means of sensitivity and specificity up to 0.976 and 0.993, respectively. These findings indicated that merely five features are able to help classify B. pseudomallei from the other groups.

## 4. Discussion

**Figure 5:**
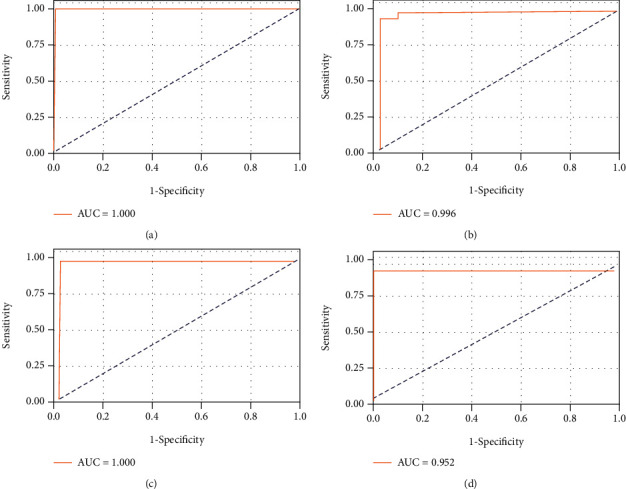
Receiver operating characteristic curves depicting SVM classification between patients with septicemic melioidosis and a mixed group including healthy donors, individuals with type 2 diabetes mellitus, individuals recovering from septicemic melioidosis, and part of other infections on testing dataset, using merely five immune cell types. Four subplots represent randomly resampling four times. AUC: area under the curve. Sensitivity and specificity were shown in the following brackets: (a) (1.000, 1.000, 1.000); (b) (0.996, 0.952, 1.000); (c) (1.000, 1.000, 0.972); (d) (0.952, 0.952, 1.000).

Bacterial blood culture is the gold standard for melioidosis diagnosis, but it has low sensitivity and takes more than four days to make definitive conclusion. That may cause the patients to miss out the best time for melioidosis treatment, according to previous studies showing that 35% of melioidosis patients died within 2 days of admission [7]. The mortality rate of the patients treated with melioidosis-appropriate drug regimens within two days of admission was decreased by 30% [8]. These results suggest that incompetent early detection is a main cause of high mortality rate. To address this challenge, researchers have developed a variety of early detection approaches. But these existing techniques have their own limitations. For instance, many serological diagnostic assays have been developed for detecting specific antibodies produced in response to *B. pseudomallei* infection. But most of them are based on poorly characterized antigens and have never been subjected to large-scale critical evaluation. Of these assays, indirect haemagglutination test is the most commonly utilized. This assay on admission has a sensitivity of only 56% in Australia [29] and 73% in Thailand [10]. Moreover, 68% of the patients with test negative on admission subsequently showed seroconversion [29]. The indirect haemagglutination test fails probably because the healthy population in the endemic regions are usually exposed to *B. pseudomallei* [2, 10]. The specific antibodies against *B. pseudomallei* are generated and become detectable in the blood. As a result, a number of patients with fever are misdiagnosed with melioidosis. Conversely, some patients with melioidosis who are supposed to produce the antibodies do not otherwise mount an adequate humoral immune response. Of note, our approach is not dependent upon the performance of humoral immune response. Removal of B cells and plasma cells did not harm the SVM model accuracy but rather improved its performance (Figure 5). Another popular serological assay is serum IgG ELISAs [30, 31]. This assay shows 72% to 83% sensitivity and greater than 95% specificity, but it is impossible to separate recovered patients from those with active infections [11, 30]. In contrast, our approach is fully capable of addressing this challenge and offers perfect sensitivity and specificity (0.988 and 1.000) (Figure 2). Lastly, it should be noted that a protein microarray using both recombinant and purified B. pseudomallei proteins for serum IgG capture shows greater than 80% sensitivity and 97% specificity [32]. Obviously, it is outperformed by our approach. Apart from the detection performance, the protein microarray-based approach is unlikely to be inexpensive in the short run.

Our findings indicated that five immune cell types (CD8+ T cells, resting CD4+ memory T cells, monocytes, M2 macrophages, and activated mast cells) were relevant to detection of septicemic melioidosis, suggesting that these cell types might be implicated in the onset and development of the disease. Similarly, in the C57BL/6 mice cell model, PECM (peritoneal exudate cells macrophage identified by nonspecific esterase) and NAPEC (nonadherent peritoneal exudate cells that were believed to be full of lymphocytes) cultures exhibited remarkably lower microbicidal activity against *B. pseudomallei*, when compared to PEC (peritoneal exudate cells) suggesting that lymphocyte, in concert with macrophages, may promote killing of *B. pseudomallei* [33]. Jenjaroen et al. found that the patients with acute melioidosis survived, with a significant change in the levels of CD4 + and CD8 + T cells, when compared to those dead [18]. Due to the technical limitations, these studies have necessarily been limited to a very narrow view of the immune response, only investigating a handful of immune cells. Moreover, they do not specify which T cell subsets play important roles in melioidosis. According to the data of CIBERSORT and SVM analysis, we propose that CD8+ T cells and resting CD4+ memory T cells may be relevant to septicemic melioidosis. In our future investigations, we will confirm the functional contributions of the two T cell subsets during septicemic melioidosis.

Macrophages present in several forms, including proinflammatory (M1), nonactivated (M0), or anti-inflammatory (M2) that play distinct roles in the initiation and development of inflammation. In melioidosis, little is known about M2 macrophages. But there are several studies of macrophage forms on the other intracellular bacterial pathogens (IBPs). For instance, M2 macrophages were a comfortable replication niche for *Salmonella* and *Brucella* strains [34, 35]. And these cells favor expansion of *Chlamydia pneumoniae* [36]. In this study, M2 macrophage was selected from 22 immune cell types, suggesting that M2 macrophage may be relevant to septicemic melioidosis. The specific role of M2 macrophage is needed to be further investigated.

Monocytes are a type of leukocyte or white blood cell. They are the largest type of leukocyte and can differentiate into macrophages and myeloid lineage dendritic cells. It was found that monocytes of patients with melioidosis well suited for *B. pseudomallei* survival and expansion, when compared to those from healthy donors [37]. In the C57BL/6 melioidosis mouse model, the role of monocyte has been elucidated. Briefly, microabscesses developed secondary to primary foci via hematogenous routes, and *B. pseudomallei* was steadily harbored inside monocytes [38, 39] located in the inflamed liver and spleen during early infection. Subsequently, the bacterium was released into the blood by the infected monocyte-derived Kupffer cells. Once bacteremia recurred or persisted, *B. pseudomallei* invaded differentiated monocytes. During late infection, the circulating monocytes harboring *B. pseudomallei* were expanded [40]. Consistent with these findings, our results revealed that monocyte was one of the most important immune cell types during septicemic melioidosis. Unexpectedly, neutrophil is not in our shortlist, while activated mast cells were included. These findings are needed to be further confirmed with experiments in the near future. Meanwhile, considering that the role of mast cell in melioidosis remains unclear, we presume that it might be a reasonable direction to investigate melioidosis.

In conclusion, the results of the present study indicate that an SVM model with five features including CD8+ T cells, resting CD4+ memory T cells, monocytes, M2 macrophages, and activated mast cells is a robust detection tool for septicemic melioidosis. As a promising diagnostic approach, it may serve as a complementary to improve detection and diagnosis of this disease.

## 5. Conclusion

It has been reported that 35% of melioidosis patients died within 2 days of admission. However, the bacterial blood culture, which is the gold standard for melioidosis diagnosis, take more than four days, along with low sensitivity, and patients could miss out the best time for melioidosis treatment. Therefore, more and more techniques for the early detection of melioidosis have been developed. However, these techniques are either costly, time-consuming, or poor performance with low sensitivity and specificity. Here, we developed a novel early detection of melioidosis, based upon the support vector machine algorithms and peripheral blood microarray data. This technique gave an excellent detection outcome with high sensitivity and specificity over other existing counterparts. More importantly, it triangulated the five immune cell types, and these cell types might be unitized to develop low-cost fast detection techniques in the near future.

## Data Availability

The datasets in this study can be obtained in NCBI datasets. The accession numbers can be found in this paper.
